# Age at menarche and depression: results from the NHANES 2005–2016

**DOI:** 10.7717/peerj.7150

**Published:** 2019-06-13

**Authors:** Yun Shen, Deepthi S. Varma, Yi Zheng, Jenny Boc, Hui Hu

**Affiliations:** 1 Department of Pharmaceutical Outcomes and Policy, University of Florida, Gainesville, FL, United States of America; 2 Department of Epidemiology, University of Florida, Gainesville, FL, United States of America; 3 College of Nursing, University of Florida, Gainesville, FL, United States of America

**Keywords:** Early menarche, NHANES, Depression

## Abstract

**Objective:**

The association between early age at menarche and depression among adolescent girls and adult women has been examined in many studies. However, inconsistent results and limitations such as small sample size, low generalizability, and measurement error exist. We aimed to address these issues to assess the association between age at menarche and depressive symptoms in a nationally representative sample of US women aged 18 years and older.

**Methods:**

We used the 2005–2016 National Health and Nutrition Examination Survey (NHANES) data with a total of 15,674 women aged 18 years and older included in our study. Logistic regression models were used after adjusting for sociodemographic and health-related factors.

**Results:**

The crude-adjusted model suggests that women with early age of menarche had 1.36 (95% CI [1.16–1.61]) times the odds of current depressive symptoms compared with the normal menarche group, after controlling for age, race/ethnicity, education, poverty income ratio (PIR) and marital status. In the fully-adjusted model, women with early menarche had 1.25 (95% CI [1.05–1.48]) times the odds of current depressive symptoms, after additionally adjusting for smoking status and body mass index (BMI). However, no significant difference was observed between the normal and late menarche groups.

**Conclusion:**

Further studies are warranted to determine the causal relationship and mechanisms between early menarche and increased risk of depression.

## Introduction

Depressive disorders have become the third leading cause of the global disease burden (Institute for Health Metrics and Evaluation, 2018), with an estimated of 322 million people suffer from depressive disorders in 2015 globally (World Health Organization, 2017). A recent study from the US reported that 8.1% of adults aged 20 or older had depressive symptoms in any given two-week period during 2013–2016 (Brody, Pratt & Hughes, 2018).

Epidemiological studies in recent years have identified substantial gender-related differences in depression regarding prevalence, incidence, course, symptomatology and risk factors (Girgus & Yang, 2015; Parker & Brotchie, 2010). Women are about twice as likely as men to be diagnosed with depressive disorders and much more likely to exhibit depressive symptoms than men. Such increased risk is particularly associated with women’s reproductive years (Lokuge et al., 2011; Steiner, Dunn & Born, 2003). Before puberty, depression rates remain similar between women and men. However, at menarche, women’s bodies undergo a sudden change in the levels of estrogen and other sex steroids that are known to be associated with depression (Bloch et al., 2000; Deecher et al., 2008; Freeman et al., 2004; Sacher et al., 2010; Schmidt et al., 1998; Smith et al., 2004; Soares & Zitek, 2008). Estrogen is likely one of the factors that leads to an increased risk of depression in women, in addition to environmental, psychosocial, behavioral, and psychological factors (Soares & Zitek, 2008; Steiner, Dunn & Born, 2003), such as obesity (Luppino et al., 2010) and cigarette smoking (Paperwalla et al., 2004).

Age at menarche is often used as a marker of female sexual maturation in epidemiological studies (Karapanou & Papadimitriou, 2010). Early menarche commonly occurs at less than 12 years old (Boden, Fergusson & Horwood, 2011; Herva et al., 2004; Joinson et al., 2013; Joinson et al., 2011; Shen et al., 2017; Stice, Presnell & Bearman, 2001), which has been associated with many health problems such as diabetes (He et al., 2009; Lakshman et al., 2008), breast cancer (Collaborative Group on Hormonal Factors in Breast Cancer, 2012), obesity (Prentice & Viner, 2013), cardiovascular disease (Lakshman et al., 2009), and psychological disorders (Kaltiala-Heino et al., 2003; Posner, 2006). Furthermore, early menarche has been shown to be associated with younger age at first sexual intercourse and first childbirth (Udry, 1979), which are known risk factors for depression (Gibbs et al., 2012; Jamieson & Wade, 2011).

Multiple studies in the past decade have examined the association between early age of menarche and other markers of pubertal timing and increased risk of depression in adolescent girls (Alcalá-Herrera & Marván, 2014; Boden, Fergusson & Horwood, 2011; Galvao et al., 2014; Ge, Conger & Elder Jr, 2001; Herva et al., 2004; Joinson et al., 2013; Joinson et al., 2011; Jung, Shin & Kang, 2015; Lien, Haavet & Dalgard, 2010; Sequeira et al., 2017; Stice, Presnell & Bearman, 2001; Trépanier et al., 2013). A recent Mendelian randomization study suggested a potential causal effect of early menarche and depressive symptoms at age 14 (Sequeira et al., 2017). However, several limitations exist in these studies. First, many previous studies were school-based and suffered from substantial selection and attrition bias (Galvao et al., 2014). Second, most studies focused on adolescence and have a short follow-up time. It is possible that the association between early menarche and depression will be different as the participants get older. Contradictory findings were observed among the few population-based studies that have examined depression among adults. Mendle, Ryan & McKone (2017) found that earlier ages at menarche were associated with higher rates of depressive symptoms in early-middle adulthood, while no significant association was observed in two other studies (Herva et al., 2004; Opoliner et al., 2014). In addition, inverse association was found in another study which focused on postmenopausal women in Korea (Jung, Shin & Kang, 2015).

Given the inconsistent results between the timing of menarche and depressive symptoms in adulthood and other limitations of the previous studies, further studies on the association between age at menarche and depression in a broad age range are warranted. The aim of this study is to use the 2005–2016 National Health and Nutrition Examination Survey (NHANES) data to examine whether age at menarche is associated with depressive symptoms in a nationally representative sample of US women aged 18 years and older.

## Materials and Methods

### Study population

The NHANES is a nationally representative cross-sectional survey that collects information among non-institutionalized civilian US citizens. NHANES are conducted on a continuous basis with data releases every 2 years since 1999. The survey employs a multi-stage probabilistic design to collect a wide range of health information through household interviews and physical examinations. In this study, we included 18,002 women aged 18 years and older who responded to the reproductive health question on the age of menarche at the Mobile Examination Center (MEC) from NHANES 2005–2016. Among them, women who had missing information on age of menarche (*n* = 2,177) were excluded. Current depressive symptoms were assessed using a nine-item screening instrument among all women aged 18 years and older, and we further excluded women with missing data on depression screening (*n* = 151), which left us with a total sample size of 15,674.

### Outcome assessment

Depressive symptoms was assessed by the Patient Health Questionnaire (PHQ-9), which is a nine-item screening instrument on depressive symptoms in the past 2 weeks with scores ranging from 0 to 3 for each item (0: not at all, 1: several days, 2: more than half the days, 3: nearly every day) and has an excellent reliability (Cronbach’s *α* over 0.85) (Kroenke, Spitzer & Williams, 2001). The total score was based on the sum of points ranging from 0 to 27. Participants were asked “over the last two weeks, how often have you been bothered by the following problems”. The nine diagnostic items include “having little interest or pleasure in doing things”, “feeling down, depressed, or hopeless”, “trouble in sleeping or sleeping too much”, “feeling tired or having little energy”, “poor appetite or overeating”, “feeling bad about yourself”, “trouble in concentrating on things”, “moving or speaking slowly or too fast”, and “thoughts that you would be better off dead or of hurting yourself”. Participants were categorized as having depression (PHQ-9 scores ≥ 10) or no depression (PHQ-9 scores < 10) using a cut-point of 10 (Kroenke & Spitzer, 2002; Manea, Gilbody & McMillan, 2012).

### Age at Menarche

Age at menarche was assessed during the MEC interview. Women were asked “how old were you when first menstrual period occurred?” We categorized women into three groups based on their age at menarche (Boden, Fergusson & Horwood, 2011; Herva et al., 2004; Joinson et al., 2013; Joinson et al., 2011; Shen et al., 2017; Stice, Presnell & Bearman, 2001): the early menarche group (<12 years old), the normal menarche group (12–13 years old), and the late menarche group (≥14 years old). We also treated age at menarche as a continuous variable in the analyses.

### Covariates

A number of potential confounders were included in this analysis based on previous literature and availability of data in the NHANES: (a) demographic and socioeconomic status including age (<30, 30–39, 40–49, 50–59, 60–69, ≥70 years old, or missing), race/ethnicity (non-Hispanic white, non-Hispanic black, or Hispanic and others), education (<high school, high school, >high school, or missing), poverty income ratio (PIR; <1.0, 1.0–2.0, ≥2.0, or missing), marital status (married, not married, or missing), (b) smoking status (current smoker, former smoker, non-smoker, or missing), (c) body mass index (BMI) (<18.5, 18.5–25.0, 25.0–30.0, 30.0–35.0, ≥35.0, or missing), and (d) regular periods in the past year (yes, no, or missing).

### Statistical analysis

Descriptive analyses were performed to assess the distribution of participants’ demographic and socioeconomic status and characteristics between women with and without depression. Logistic regression models were used to explore the associations between age at menarche and depression, and odds ratios (ORs) and 95% confidence intervals (CIs) were obtained. We used a crude-adjusted model controlling for age, race/ethnicity, education, PIR and marital status, and a fully-adjusted model additionally controlling for smoking status, BMI, and regular periods in the past year. To account for the potential concerns that retrospective report of age of menarche may be less reliable for older women and older women may have greater emotion regulation (Carstensen, Fung & Charles, 2003), we also examined whether potential interaction exists between age and the timing of menarche. To ensure the correct estimation of sampling error, the sample weights, stratification and clustering design variables were accounted for in the analyses. Following the NHANES analytic and reporting guidelines, a twelve-year MEC subsample weight was calculated for the combined data of 2005–2016 by assigning one-sixth of the subsample weight for each 2-year data cycle. To account for the potential bias created by including participants with non-missing information on age at menarche and current depressive symptoms, we conducted multiple imputations for all missing data using chained equations. All covariates as well as exposure and outcome variables were included in the imputation process, and 50 imputed data sets were generated. All statistical analyses were conducted using the survey package in R 3.4.4. The study has been approved by the Institutional Review Board at University of Florida (IRB201900509).

**Table 1:** Characteristics of women aged 18 years and older by current depressive symptoms in NHANES 2005–2016 (*n* = 15,674).

	With current depressive symptoms (*n*= 1,705)		No current depressive symptoms (*n*= 13,969)		Total	
	*N*	Percent (95% CI)^a^	*N*	Percent (95% CI)^a^	*N*	Percent (95% CI)^a^
Age at menarche (years)						
Early (<12)	455	27.5 (25.2, 30.0)	2,941	19.9 (19.0, 20.9)	7,830	51.6 (50.6, 52.7)
Normal (12–13)	820	48.1 (44.7, 51.5)	7,010	52.0 (50.9, 53.2)	3,396	20.7 (19.7, 21.6)
Late (≥14)	430	24.4 (21.8, 27.1)	4,018	28.0 (26.9, 29.2)	4,448	27.7 (26.6, 28.8)
Age (years)						
<30	312	18.1 (15.7, 20.9)	3,234	20.8 (19.6, 22.0)	3,546	20.5 (19.4, 21.7)
30–39	277	17.3 (15.0, 19.8)	2,141	16.4 (15.5, 17.3)	2,418	16.5 (15.6, 17.4)
40–49	326	22.5 (19.9, 25.3)	2,257	18.3 (17.3, 19.2)	2,583	18.7 (17.8, 19.6)
50–59	342	20.8 (18.2, 23.7)	1,954	17.5 (16.7, 18.4)	2,296	17.9 (17.0, 18.8)
60–69	277	12.8 (10.9, 15.0)	2,177	13.6 (12.8, 14.3)	2,454	13.5 (12.8, 14.3)
≥70	171	8.4 (7.0, 10.2)	2,206	13.5 (12.6, 14.4)	2,377	13.0 (12.2, 13.8)
Race/ethnicity						
Non-Hispanic White	677	62.9 (59.5, 67.1)	5,925	68.8 (65.8, 71.5)	6,602	68.2 (65.3, 71.0)
Non-Hispanic Black	385	14.8 (12.6, 17.3)	3,027	11.6 (10.0, 13.4)	3,412	11.9 (10.3, 13.7)
Hispanic and others	643	22.2 (19.0, 25.9)	5,017	19.6 (17.8, 21.6)	5,660	19.9 (18.0, 21.9)
Education						
<High school	630	27.0 (24.3, 29.9)	3,242	15.0 (13.8, 16.2)	3,872	16.1 (14.9, 17.4)
High school	386	25.4 (22.8, 28.1)	3,196	21.8 (20.7, 23.0)	3,582	22.2 (21.1, 23.3)
>High school	688	47.6 (44.0, 51.2)	7,521	63.2 (61.3, 65.0)	8,209	61.7 (59.8, 63.5)
Missing	1	0.0 (0.0, 0.1)	10	0.0 (0.0, 0.1)	11	0.0 (0.0, 0.1)
PIR						
<1.0	644	30.0 (26.9, 33.2)	2,790	13.0 (12.0, 14.0)	3,434	14.6 (13.5, 15.7)
1.0–2.0	486	27.4 (24.6, 30.4)	3,344	19.2 (18.3, 20.2)	3,830	20.0 (19.0, 21.1)
≥2.0	433	36.4 (32.2, 40.9)	6,734	61.7 (59.9, 63.5)	7,167	59.3 (57.4, 61.2)
Missing	142	6.2 (4.9, 7.6)	1,101	6.1 (5.5, 6.7)	1,243	6.1 (5.5, 6.7)
Marital status						
Married	542	35.5 (33.0, 38.2)	6,430	53.0 (51.5, 54.5)	6,972	51.4 (49.9, 52.9)
Not married	1,107	62.5 (59.9, 65.1)	6,934	44.4 (42.9, 45.8)	8,041	46.1 (44.6, 47.6)
Missing	56	2.0 (1.3, 2.8)	605	2.6 (2.3, 2.9)	661	2.5 (2.2, 2.8)
Smoking status						
Current smoker	585	38.3 (35.0, 41.7)	2,021	15.6 (14.7, 16.7)	2,606	17.8 (16.8, 18.9)
Former smoker	302	18.6 (15.8, 21.7)	2,505	20.8 (19.6, 22.0)	2,807	20.6 (19.4, 21.8)
Non-smoker	768	41.7 (38.3, 45.2)	8,858	61.5 (60.1, 62.8)	9,626	59.6 (58.3, 60.9)
Missing	50	1.5 (0.9, 2.2)	585	2.1 (1.8, 2.4)	635	2.0 (1.7, 2.3)
BMI						
<18.5	35	2.2 (1.4, 3.5)	298	2.1 (1.8, 2.4)	333	2.1 (1.8, 2.4)
18.5–25.0	371	21.4 (18.9, 24.1)	4,192	33.2 (31.9, 34.6)	3,463	32.1 (30.9, 33.4)
25.0–30.0	394	23.6 (21.0, 26.5)	3,935	28.0 (26.9, 29.2)	4,329	27.6 (26.5, 28.8)
30.0–35.0	386	22.2 (19.5, 25.2)	2,729	17.9 (17.2, 18.7)	3,115	18.4 (17.6, 19.1)
≥35.0	496	29.3 (26.4, 32.4)	2,669	17.9 (16.9, 18.9)	3,165	19.0 (18.1, 20.0)
Missing	23	1.3 (0.8, 2.1)	146	0.7 (0.6, 1.0)	169	0.8 (0.6, 1.0)
Regular periods in the past year						
Yes	818	50.7 (47.4, 54.1)	7,172	52.0 (50.4, 54.0)	7,990	51.9 (50.4, 53.5)
No	885	49.2 (45.8, 52.6)	6,794	47.9 (46.3, 49.5)	7,679	48.0 (46.5, 49.6)
Missing	2	0.1 (0.0, 0.4)	3	0.0 (0.0, 0.2)	5	0.0 (0.0, 0.4)

**Notes.**

a Weighted percentage with 95% confidence interval.

## Results

Table 1 shows the characteristics of women by current depressive symptoms. Women with current depressive symptoms were more likely to be current smokers (38.3% vs 15.6%), with BMI ≥35.0 (29.3% vs 17.9%) and had early age of menarche (27.5% vs 19.9%) than those without current depressive symptoms. Women with current depressive symptoms were also more likely to be un-married (62.5% vs 44.4%) and to have less than high school (27.0% vs 15.0%) or high school level education (25.4% vs 21.8%) and PIR levels <1.0 (30.0% vs 13.0%) or 1.0–2.0 (27.4% vs 19.2%). Women without current depressive symptoms were more likely to be 70 years and older (13.5% vs 8.4%) and have a normal BMI (33.2% vs 21.4%) compared with those with current depressive symptoms.

Table 2 provides the unadjusted, crude-adjusted and fully-adjusted ORs from the logistic regression models assessing the associations between age at menarche and current depressive symptoms (Tables S1 and S2 shows the ORs and 95% CIs for covariates). After controlling for age, race/ethnicity, education, PIR and marital status, the crude-adjusted model showed that women with early age at menarche had 1.36 (95% CI [1.16–1.61]) times the odds of current depressive symptoms compared with the normal menarche group. However, no significant difference was observed between the late menarche group and the normal group. Consistent results were also found in the fully-adjusted model after additionally adjusting for smoking status, BMI, and regular periods in the past year. Compared with women who had normal menarche age, women with early menarche had 1.25 (95% CI [1.05–1.48]) times the odds of current depressive symptoms, while no significant difference was observed between the normal and late menarche groups. When treated as a continuous variable, each 1-year decrease in age of menarche is associated with a significant increase in the odds of current depressive symptoms (adjusted OR: 1.05, 95% CI [1.01–1.09]). No significant interaction between age at menarche and age at interview was found. Consistent results were observed from the multiple imputations as shown in Table S3.

**Table 2:** ORs (95% CIs) of menarche age associated with current depressive symptoms in NHANES 2005–2016 (*n* = 15,674).

Age at menarche	Unadjusted model	Crude-adjusted model^a^	Fully-adjusted model^b^
	OR (95% CI)	OR (95% CI)	OR (95% CI)
Categorical^c^			
Normal	Reference	Reference	Reference
Early	1.49 (1.29, 1.73)	1.36 (1.16, 1.61)	1.27 (1.08, 1.50)
Late	0.94 (0.78, 1.12)	0.89 (0.74, 1.07)	0.98 (0.81, 1.19)
Continuous^d^	1.10 (1.06, 1.14)	1.09 (1.05, 1.13)	1.05 (1.01, 1.09)

**Notes.**

a Adjusted for age, race/ethnicity, education, PIR, marital status.

b Adjusted for age, race/ethnicity, education, PIR, marital status, smoking status, BMI, and regular periods in the past year.

c Normal: 12–13 years; Early: <12 years; Late: ≥14 years.

d Each 1-year decrease in age of menarche.

## Discussion

Using a nationally representative sample of the US population, we found that early menarche is associated with current depressive symptoms. The observed association persisted after adjusting for potential confounders such as age, race/ethnicity, education, PIR, marital status, smoking status and BMI. The results observed in this study are consistent with several previous studies. Multiple studies have shown that early age at menarche or early pubertal timing is associated with an increased risk of depression in adolescent girls (Alcalá-Herrera & Marván, 2014; Boden, Fergusson & Horwood, 2011; Galvao et al., 2014; Ge, Conger & Elder Jr, 2001; Joinson et al., 2011; Lien, Haavet & Dalgard, 2010; Nolen-Hoeksema & Girgus, 1994; Patton et al., 1996; Stice, Presnell & Bearman, 2001; Trépanier et al., 2013). Tondo et al. (2017) found the association between age at menarche and age at onset of depression. Mendle, Ryan & McKone (2017) also found that early menarche was associated with an increased risk of depressive symptoms and antisocial behavior in early to middle adulthood. However, this is the first study to find a significant relationship between early menarche and depressive symptoms in adulthood with a broad age range using a nationally representative sample (NHANES 2005–2016). These findings support the hypothesis that early menarche could be used as one of the markers to identify adolescents who have a higher risk of developing depressive symptoms in the future. Hormonal, neurocognitive and psychosocial factors could be reasons for this association. The onset of puberty increases hormonal levels which results in a rapid fluctuation in the estrogen production in a woman’s body. The inability to adapt to these rapid changes could make women susceptible to depression (Sequeira et al., 2017). Further, inconsistency between levels of biological and cognitive maturation, and feeling ‘different’ than one’s peers, may also cause psychological distress and depressive symptoms during early adolescent years (Holder & Blaustein, 2014).

Although there are accumulating evidences supporting the assertion that early menarche is a risk factor for psychopathology among adolescent girls, the mechanisms underlying the gender-related differences in puberty age and onset of depressive symptoms are still unclear in adulthood. For instance, a recent study from the UK reported that while early menarche had elevated the risk of depression among early to middle adolescence, there was no association observed between the timing of menarche and depressive symptoms in later adolescence and young adulthood (Joinson et al., 2013). Similarly, in the Nurses’ Health Study II, (Opoliner et al., 2014) there was no association found between early or late menarche and depressive symptoms in young adulthood. Several limitations in these previous studies may have led to inconsistent findings. These contradictions could be due to the use of different types of instruments to assess depression in these studies. Secondly, none of these studies used population-representative samples. For example, most of the women included in the Nurse’s Health Study were non-Hispanic White, which may lead to substantial selection bias and thus the results may not be generalizable to the total population.

This study has several strengths. The NHANES data provided us with a unique opportunity to study the association between age at menarche and depressive symptoms in a large multiethnic, nationally representative sample of the US population. In addition, we were also able to adjust for a wide range of potential confounders such as sociodemographic characteristics, BMI, and cigarette smoking to assess the true association between age at menarche and depressive symptoms. There are several limitations need to be noted. First, the self-reported information of age at menarche may introduce potential misclassification bias. However, this is likely to be a non-differential bias, which will bias the findings to null. Second, age at menarche is only one variable that reflects the timing of puberty. Future studies with more information on the timing and tempo of pubertal development are needed. Third, the dataset did not include information on potential confounders such as the family history of depression. Furthermore, we relied on a screening measure of depression which only assessed depressive symptoms in the past two weeks, and past histories of depressive episodes or clinical diagnoses of depression are not available. Future studies with longitudinal data on history of recurrent depressive episodes are warranted to further confirm the findings.

## Conclusions

In a nationally representative sample of US adults, early menarche was found to be associated with current depressive symptoms (assessed using the PHQ-9) after adjusting for confounding factors, such as age, race/ethnicity, education, poverty income ratio, marital status, cigarette smoking, and BMI. Further studies are warranted to determine the causal relationship and mechanisms between early menarche and increased risk of depression.

##  Supplemental Information

10.7717/peerj.7150/supp-1 Table S1 ORs (95% CIs) of menarche age (categorical) and covariates associated with current depressive symptoms in NHANES 2005–2016 (*n* = 15,674) The ORs and 95% CIs for covariates from the models where age at menarche was treated as a categorical variable.

10.7717/peerj.7150/supp-2 Table S2 ORs (95% CIs) of menarche age (continuous) and covariates associated with current depressive symptoms in NHANES 2005–2016 (*n* = 15,674) The ORs and 95% CIs for covariates from the models where age at menarche was treated as a continuous variable.

10.7717/peerj.7150/supp-3 Table S3 Results from the multiple imputations (*n* = 18,002) The results from the multiple imputations, which are consistent with the findings from the main analyses.

10.7717/peerj.7150/supp-4 Supplemental Information 1 R codes to access, engineer, and analyze data from the NHANES R codes used in this study to access, engineer, and analyze data from the NHANES.

## Additional Information and Declarations

### Data Availability

The following information was supplied regarding data availability: The R codes are available in the Supplemental File. The raw data can be accessed through the R codes in the Supplemental Files or from the NHANES website: https://wwwn.cdc.gov/nchs/nhanes/Default.aspx.

## References

[ref-1] Alcalá-Herrera V, Marván ML (2014). Early menarche, depressive symptoms, and coping strategies. Journal of Adolescence.

[ref-2] Bloch M, Schmidt PJ, Danaceau M, Murphy J, Nieman L, Rubinow DR (2000). Effects of gonadal steroids in women with a history of postpartum depression. American Journal of Psychiatry.

[ref-3] Boden JM, Fergusson DM, Horwood LJ (2011). Age of menarche and psychosocial outcomes in a New Zealand birth cohort. Journal of the American Academy of Child & Adolescent Psychiatry.

[ref-4] Brody DJ, Pratt LA, Hughes JP (2018). Prevalence of depression among adults aged 20 and over: United States, 2013–2016. NCHS Data Brief.

[ref-5] Carstensen LL, Fung HH, Charles ST (2003). Socioemotional selectivity theory and the regulation of emotion in the second half of life. Motivation and Emotion.

[ref-6] Collaborative Group on Hormonal Factors in Breast Cancer (2012). Menarche, menopause, and breast cancer risk: individual participant meta-analysis, including 118,964 women with breast cancer from 117 epidemiological studies. The Lancet Oncology.

[ref-7] Deecher D, Andree TH, Sloan D, Schechter LE (2008). From menarche to menopause: exploring the underlying biology of depression in women experiencing hormonal changes. Psychoneuroendocrinology.

[ref-8] Freeman EW, Sammel MD, Liu L, Gracia CR, Nelson DB, Hollander L (2004). Hormones and menopausal status as predictors of depression in womenin transition to menopause. Archives of General Psychiatry.

[ref-9] Galvao TF, Silva MT, Zimmermann IR, Souza KM, Martins SS, Pereira MG (2014). Pubertal timing in girls and depression: a systematic review. Journal of Affective Disorders.

[ref-10] Ge X, Conger RD, Elder Jr GH (2001). Pubertal transition, stressful life events, and the emergence of gender differences in adolescent depressive symptoms. Developmental Psychology.

[ref-11] Gibbs CM, Wendt A, Peters S, Hogue CJ (2012). The impact of early age at first childbirth on maternal and infant health. Paediatric and Perinatal Epidemiology.

[ref-12] Girgus JS, Yang K (2015). Gender and depression. Current Opinion in Psychology.

[ref-13] He C, Zhang C, Hunter DJ, Hankinson SE, Buck Louis GM, Hediger ML, Hu FB (2009). Age at menarche and risk of type 2 diabetes: results from 2 large prospective cohort studies. American Journal of Epidemiology.

[ref-14] Herva A, Jokelainen J, Pouta A, Veijola J, Timonen M, Karvonen JT, Joukamaa M (2004). Age at menarche and depression at the age of 31 years: findings from the Northern Finland 1966 birth cohort study. Journal of Psychosomatic Research.

[ref-15] Holder MK, Blaustein JD (2014). Puberty and adolescence as a time of vulnerability to stressors that alter neurobehavioral processes. Frontiers in Neuroendocrinology.

[ref-16] Institute for Health Metrics and Evaluation (2018). Findings from the global burden of disease study 2017.

[ref-17] Jamieson LK, Wade TJ (2011). Early age of first sexual intercourse and depressive symptomatology among adolescents. Journal of Sex Research.

[ref-18] Joinson C, Heron J, Araya R, Lewis G (2013). Early menarche and depressive symptoms from adolescence to young adulthood in a UK cohort. Journal of the American Academy of Child & Adolescent Psychiatry.

[ref-19] Joinson C, Heron J, Lewis G, Croudace T, Araya R (2011). Timing of menarche and depressive symptoms in adolescent girls from a UK cohort. The British Journal of Psychiatry.

[ref-20] Jung SJ, Shin A, Kang D (2015). Menarche age, menopause age and other reproductive factors in association with post-menopausal onset depression: results from Health Examinees Study (HEXA). Journal of Affective Disorders.

[ref-21] Kaltiala-Heino R, Marttunen M, Rantanen P, Rimpelä M (2003). Early puberty is associated with mental health problems in middle adolescence. Social Science & Medicine.

[ref-22] Karapanou O, Papadimitriou A (2010). Determinants of menarche. Reproductive Biology and Endocrinology.

[ref-23] Kroenke K, Spitzer RL (2002). The PHQ-9: a new depression diagnostic and severity measure. Psychiatric Annals.

[ref-24] Kroenke K, Spitzer RL, Williams JB (2001). The PHQ-9: validity of a brief depression severity measure. Journal of General Internal Medicine.

[ref-25] Lakshman R, Forouhi N, Luben R, Bingham S, Khaw K, Wareham N, Ong K (2008). Association between age at menarche and risk of diabetes in adults: results from the EPIC-Norfolk cohort study. Diabetologia.

[ref-26] Lakshman R, Forouhi NG, Sharp SJ, Luben R, Bingham SA, Khaw K-T, Wareham NJ, Ong KK (2009). Early age at menarche associated with cardiovascular disease and mortality. The Journal of Clinical Endocrinology & Metabolism.

[ref-27] Lien L, Haavet OR, Dalgard F (2010). Do mental health and behavioural problems of early menarche persist into late adolescence? A three year follow-up study among adolescent girls in Oslo, Norway. Social Science & Medicine.

[ref-28] Lokuge S, Frey BN, Foster JA, Soares CN, Steiner M (2011). Depression in women: windows of vulnerability and new insights into the link between estrogen and serotonin. The Journal of Clinical Psychiatry.

[ref-29] Luppino FS, De Wit LM, Bouvy PF, Stijnen T, Cuijpers P, Penninx BW, Zitman FG (2010). Overweight, obesity, and depression: a systematic review and meta-analysis of longitudinal studies. Archives of General Psychiatry.

[ref-30] Manea L, Gilbody S, McMillan D (2012). Optimal cut-off score for diagnosing depression with the Patient Health Questionnaire (PHQ-9): a meta-analysis. Canadian Medical Association Journal/Journal de l’Association Medicale Canadienne.

[ref-31] Mendle J, Ryan RM, McKone KM (2017). Age at menarche, depression, and antisocial behavior in adulthood. Pediatrics.

[ref-32] Nolen-Hoeksema S, Girgus JS (1994). The emergence of gender differences in depression during adolescence. Psychological Bulletin.

[ref-33] Opoliner A, Carwile JL, Blacker D, Fitzmaurice GM, Austin SB (2014). Early and late menarche and risk of depressive symptoms in young adulthood. Archives of Women’s Mental Health.

[ref-34] Paperwalla KN, Levin TT, Weiner J, Saravay SM (2004). Smoking and depression. The Medical Clinics of North America.

[ref-35] Parker G, Brotchie H (2010). Gender differences in depression. International Review of Psychiatry.

[ref-36] Patton G, Hibbert M, Carlin J, Shao Q, Rosier M, Caust J, Bowes G (1996). Menarche and the onset of depression and anxiety in Victoria, Australia. Journal of Epidemiology & Community Health.

[ref-37] Posner RB (2006). Early menarche: a review of research on trends in timing, racial differences, etiology and psychosocial consequences. Sex Roles.

[ref-38] Prentice P, Viner RM (2013). Pubertal timing and adult obesity and cardiometabolic risk in women and men: a systematic review and meta-analysis. International Journal of Obesity.

[ref-39] Sacher J, Wilson AA, Houle S, Rusjan P, Hassan S, Bloomfield PM, Stewart DE, Meyer JH (2010). Elevated brain monoamine oxidase A binding in the early postpartum period. Archives of General Psychiatry.

[ref-40] Schmidt PJ, Nieman LK, Danaceau MA, Adams LF, Rubinow DR (1998). Differential behavioral effects of gonadal steroids in women with and in those without premenstrual syndrome. New England Journal of Medicine.

[ref-41] Sequeira M-E, Lewis SJ, Bonilla C, Smith GD, Joinson C (2017). Association of timing of menarche with depressive symptoms and depression in adolescence: Mendelian randomisation study. The British Journal of Psychiatry.

[ref-42] Shen Y, Hu H, Taylor BD, Kan H, Xu X (2017). Early menarche and gestational diabetes mellitus at first live birth. Maternal and Child Health Journal.

[ref-43] Smith LJ, Henderson JA, Abell CW, Bethea CL (2004). Effects of ovarian steroids and raloxifene on proteins that synthesize, transport, and degrade serotonin in the raphe region of macaques. Neuropsychopharmacology.

[ref-44] Soares CN, Zitek B (2008). Reproductive hormone sensitivity and risk for depression across the female life cycle: a continuum of vulnerability?. Journal of Psychiatry & Neuroscience.

[ref-45] Steiner M, Dunn E, Born L (2003). Hormones and mood: from menarche to menopause and beyond. Journal of Affective Disorders.

[ref-46] Stice E, Presnell K, Bearman SK (2001). Relation of early menarche to depression, eating disorders, substance abuse, and comorbid psychopathology among adolescent girls. Developmental Psychology.

[ref-47] Tondo L, Pinna M, Serra G, De Chiara L, Baldessarini RJ (2017). Age at menarche predicts age at onset of major affective and anxiety disorders. European Psychiatry.

[ref-48] Trépanier L, Juster R-P, Marin M-F, Plusquellec P, Francois N, Sindi S, Wan N, Findlay H, Schramek T, Andrews J (2013). Early menarche predicts increased depressive symptoms and cortisol levels in Quebec girls ages 11 to 13. Development and Psychopathology.

[ref-49] Udry JR (1979). Age at menarche, at first intercourse, and at first pregnancy. Journal of Biosocial Science.

[ref-50] World Health Organization (WHO) (2017). Depression and other common mental disorders: global health estimates.

