# Supplementary material for: Age at menarche and depression: results from the NHANES 2005–2016
Source: PeerJ. 2019 Jun 13;7:e7150. doi: 10.7717/peerj.7150 (PMC6571127; doi:10.7717/peerj.7150)
Supplement: Table S1 — The ORs and 95% CIs for covariates from the models where age at menarche was treated as a categorical variable. [file peerj-07-7150-s001.docx]

| **Supplemental Table 1.** ORs (95% CIs) of menarche age (categorical) and covariates associated with current depressive symptoms in NHANES 2005-2016 (n=15,674). | | | |
| --- | --- | --- | --- |
|  | Unadjusted model | Crude-adjusted model ^a^ | Fully-adjusted model ^b^ |
| Age at menarche | OR (95% CI) | OR (95% CI) | OR (95% CI) |
| Age at menarche ^c^ |  |  |  |
| Normal | Reference | Reference | Reference |
| Early | 1.49 (1.29, 1.73) | 1.36 (1.16, 1.61) | 1.27 (1.08, 1.50) |
| Late | 0.94 (0.78, 1.12) | 0.89 (0.74, 1.07) | 0.98 (0.81, 1.19) |
| Age (years) |  |  |  |
| <30 | - | Reference | Reference |
| 30-39 | - | 1.54 (1.19, 1.64) | 1.33 (1.02, 1.74) |
| 40-49 | - | 2.00 (1.57, 2.55) | 1.61 (1.25, 2.07) |
| 50-59 | - | 1.94 (1.54, 2.43) | 1.46 (1.10, 1.93) |
| 60-69 | - | 1.41 (1.09, 1.82) | 1.12 (0.80, 1.55) |
| ≥70 | - | 0.72 (0.53, 0.98) | 0.70 (0.47, 1.04) |
| Race/ethnicity |  |  |  |
| Non-Hispanic White | - | Reference | Reference |
| Non-Hispanic Black | - | 0.86 (0.73, 1.01) | 0.88 (0.74, 1.03) |
| Hispanic and others | - | 0.82 (0.69, 0.98) | 1.04 (0.87, 1.24) |
| Education |  |  |  |
| <High school | - | Reference | Reference |
| High school | - | 0.79 (0.66, 0.94) | 0.80 (0.67, 0.95) |
| >High school | - | 0.58 (0.47, 0.70) | 0.69 (0.57, 0.84) |
| Missing | - | 0.50 (0.05, 4.55) | 0.62 (0.06, 6.03) |
| PIR |  |  |  |
| <1.0 | - | Reference | Reference |
| 1.0-2.0 | - | 0.70 (0.59, 0.82) | 0.75 (0.64, 0.88) |
| ≥2.0 | - | 0.32 (0.27, 0.40) | 0.39 (0.32, 0.48) |
| Missing | - | 0.53 (0.42, 0.66) | 0.63 (0.50, 0.79) |
| Marital status |  |  |  |
| Married | - | Reference | Reference |
| Not married | - | 1.82 (1.61, 2.05) | 1.65 (1.46, 1.86) |
| Missing | - | 1.04 (0.68, 1.60) | 1.44 (0.95, 2.18) |
| Smoking status |  |  |  |
| Current smoker | - | - | Reference |
| Former smoker | - | - | 0.49 (0.40, 0.60) |
| Non-smoker | - | - | 0.37 (0.31, 0.44) |
| Missing | - | - | 0.36 (0.22, 0.57) |
| BMI |  |  |  |
| <18.5 | - | - | Reference |
| 18.5-25.0 | - | - | 0.88 (0.51, 1.50) |
| 25.0-30.0 | - | - | 1.08 (0.63, 1.85) |
| 30.0-35.0 | - | - | 1.51 (0.87, 2.61) |
| ≥35.0 | - | - | 1.89 (1.11, 3.20) |
| Missing | - | - | 1.90 (0.83, 4.33) |
| Regular periods in the past year |  |  |  |
| No | - | - | Reference |
| Yes | - | - | 0.83 (0.66, 1.04) |
| Missing | - | - | 2.49 (0.51, 12.17) |
|  |  |  |  |
|  |  |  |  |
|  |  |  |  |
|  |  |  |  |
| ^a^ Adjusted for age, race/ethnicity, education, PIR, marital status.  ^b^ Adjusted for age, race/ethnicity, education, PIR, marital status, smoking status, BMI, and regular periods in the past year.  ^c^ Normal: 12-13 years; Early: <12 years; Late: ≥14 years. | | | |
