# Supplementary material for: Age at menarche and depression: results from the NHANES 2005–2016
Source: PeerJ. 2019 Jun 13;7:e7150. doi: 10.7717/peerj.7150 (PMC6571127; doi:10.7717/peerj.7150)
Supplement: Table S2 — The ORs and 95% CIs for covariates from the models where age at menarche was treated as a continuous variable. [file peerj-07-7150-s002.docx]

| **Supplemental Table 2.** ORs (95% CIs) of menarche age (continuous) and covariates associated with current depressive symptoms in NHANES 2005-2016 (n=15,674). | | | |
| --- | --- | --- | --- |
|  | Unadjusted model | Crude-adjusted model ^a^ | Fully-adjusted model ^b^ |
| Age at menarche | OR (95% CI) | OR (95% CI) | OR (95% CI) |
| Age at menarche ^c^ | 1.10 (1.06, 1.14) | 1.09(1.05, 1.13) | 1.05 (1.01, 1.09) |
| Age (years) |  |  |  |
| <30 | - | Reference | Reference |
| 30-39 | - | 1.54 (1.19, 1.99) | 1.33 (1.02, 1.74) |
| 40-49 | - | 2.01 (1.57, 2.55) | 1.61 (1.26, 2.08) |
| 50-59 | - | 1.94 (1.54, 2.44) | 1.46 (1.10, 1.94) |
| 60-69 | - | 1.41 (1.09, 1.83) | 1.12 (0.80, 1.56) |
| ≥70 | - | 0.73 (0.54, 0.99) | 0.70 (0.48, 1.04) |
| Race/ethnicity |  |  |  |
| Non-Hispanic White | - | Reference | Reference |
| Non-Hispanic Black | - | 0.86 (0.73, 1.01) | 0.88 (0.74, 1.04) |
| Hispanic and others | - | 0.82 (0.69, 0.99) | 1.04 (0.87, 1.25) |
| Education |  |  |  |
| <High school | - | Reference | Reference |
| High school | - | 0.78 (0.66, 0.94) | 0.80 (0.67, 0.95) |
| >High school | - | 0.57 (0.47, 0.70) | 0.69 (0.57, 0.84) |
| Missing | - | 0.51 (0.06, 4.71) | 0.63 (0.06, 6.20) |
| PIR |  |  |  |
| <1.0 | - | Reference | Reference |
| 1.0-2.0 | - | 0.70 (0.59, 0.82) | 0.75 (0.64, 0.88) |
| ≥2.0 | - | 0.32 (0.27, 0.39) | 0.39 (0.32, 0.48) |
| Missing | - | 0.53 (0.42, 0.66) | 0.63 (0.50, 0.79) |
| Marital status |  |  |  |
| Married | - | Reference | Reference |
| Not married | - | 1.82 (1.62, 2.06) | 1.65 (1.46, 1.86) |
| Missing | - | 1.04 (0.68, 1.60) | 1.45 (0.95, 2.19) |
| Smoking status |  |  |  |
| Current smoker | - | - | Reference |
| Former smoker | - | - | 0.49 (0.40, 0.60) |
| Non-smoker | - | - | 0.37 (0.31, 0.440 |
| Missing | - | - | 0.35 (0.22, 0.57) |
| BMI |  |  |  |
| <18.5 | - | - | Reference |
| 18.5-25.0 | - | - | 0.88 (0.51, 1.51) |
| 25.0-30.0 | - | - | 1.09 (0.64, 1.85) |
| 30.0-35.0 | - | - | 1.52 (0.88, 2.62) |
| ≥35.0 | - | - | 1.90 (1.12, 3.21) |
| Missing | - | - | 1.89 (0.83, 4.32) |
| Regular periods in the past year |  |  |  |
| No | - | - | Reference |
| Yes | - | - | 0.83 (0.66, 1.04) |
| Missing | - | - | 2.67 (0.52, 13.67) |
| ^a^ Adjusted for age, race/ethnicity, education, PIR, marital status.  ^b^ Adjusted for age, race/ethnicity, education, PIR, marital status, smoking status, BMI, and regular periods in the past year. ^c^ Each 1-year decrease in age of menarche. | | | |
