# Supplementary material for: Age at menarche and depression: results from the NHANES 2005–2016
Source: PeerJ. 2019 Jun 13;7:e7150. doi: 10.7717/peerj.7150 (PMC6571127; doi:10.7717/peerj.7150)
Supplement: Table S3 — The results from the multiple imputations, which are consistent with the findings from the main analyses. [file peerj-07-7150-s003.docx]

| **Supplemental Table 3.** Results from the multiple imputations (n=18,002). | | |
| --- | --- | --- |
|  | Original results ^a^ | Multiple imputation ^a^ |
| Age at menarche | OR (95% CI) | OR (95% CI) |
| Categorical ^b^ |  |  |
| Normal | Reference | Reference |
| Early | 1.27 (1.08, 1.50) | 1.26 (1.08, 1.47) |
| Late | 0.98 (0.81, 1.19) | 0.97 (0.82, 1.15) |
| Continuous ^c^ | 1.05 (1.01, 1.09) | 1.04 (1.01, 1.08) |
| ^a^ Adjusted for age, race/ethnicity, education, PIR, marital status, smoking status, BMI, and regular periods in the past year.  ^b^ Normal: 12-13 years; Early: <12 years; Late: ≥14 years. ^c^ Each 1-year decrease in age of menarche. | | |
