# Supplementary material for: Age at menarche and depression: results from the NHANES 2005–2016
Source: PeerJ. 2019 Jun 13;7:e7150. doi: 10.7717/peerj.7150 (PMC6571127; doi:10.7717/peerj.7150)
Supplement: Supplemental Information 1 — R codes used in this study to access, engineer, and analyze data from the NHANES. [file peerj-07-7150-s004.docx]

**Supplemental File.** R codes to access, engineer, and analyze data from the NHANES.

library(nhanesA)

library(sqldf)

library(dplyr)

library(survey)

options(survey.lonely.psu="adjust")

library(gtools)

library(DescTools)

library(tableone)

library(mitools)

library(mice)

#Get Relevant Tables(2005-2016)

#Demographics

DEMO_D<-nhanes("DEMO_D")

DEMO_E<-nhanes("DEMO_E")

DEMO_F<-nhanes("DEMO_F")

DEMO_G<-nhanes("DEMO_G")

DEMO_H<-nhanes("DEMO_H")

DEMO_I<-nhanes("DEMO_I")

#questionaire data-depression

DPQ_D<-nhanes("DPQ_D")

DPQ_E<-nhanes("DPQ_E")

DPQ_F<-nhanes("DPQ_F")

DPQ_G<-nhanes("DPQ_G")

DPQ_H<-nhanes("DPQ_H")

DPQ_I<-nhanes("DPQ_I")

#questionaire data-reproductive data

RHQ_D<-nhanes("RHQ_D")

RHQ_E<-nhanes("RHQ_E")

RHQ_F<-nhanes("RHQ_F")

RHQ_G<-nhanes("RHQ_G")

RHQ_H<-nhanes("RHQ_H")

RHQ_I<-nhanes("RHQ_I")

#questionaire data-smoking-cigarette use

SMQ_D<-nhanes("SMQ_D")

SMQ_E<-nhanes("SMQ_E")

SMQ_F<-nhanes("SMQ_F")

SMQ_G<-nhanes("SMQ_G")

SMQ_H<-nhanes("SMQ_H")

SMQ_I<-nhanes("SMQ_I")

#examination data-body measures

BMX_D<-nhanes("BMX_D")

BMX_E<-nhanes("BMX_E")

BMX_F<-nhanes("BMX_F")

BMX_G<-nhanes("BMX_G")

BMX_H<-nhanes("BMX_H")

BMX_I<-nhanes("BMX_I")

#questionaire data-medical condition

MCQ_D<-nhanes("MCQ_D")

MCQ_E<-nhanes("MCQ_E")

MCQ_F<-nhanes("MCQ_F")

MCQ_G<-nhanes("MCQ_G")

MCQ_H<-nhanes("MCQ_H")

MCQ_I<-nhanes("MCQ_I")

#questioniaire-diabete

DIQ_D<-nhanes("DIQ_D")

DIQ_E<-nhanes("DIQ_E")

DIQ_F<-nhanes("DIQ_F")

DIQ_G<-nhanes("DIQ_G")

DIQ_H<-nhanes("DIQ_H")

DIQ_I<-nhanes("DIQ_I")

###############

#Data cleaning#

###############

#Demographics

DEMO_D[]<- lapply(DEMO_D, as.numeric)

q<-'SELECT SEQN,RIAGENDR,RIDAGEYR,RIDRETH1,DMDBORN AS DMDBORN4,DMDEDUC3,DMDEDUC2,DMDMARTL,INDFMPIR,WTMEC2YR,SDMVPSU,SDMVSTRA

FROM DEMO_D

;'

DEMO_D<-sqldf(q)

DEMO_E[]<- lapply(DEMO_E, as.numeric)

q<-'SELECT SEQN,RIAGENDR,RIDAGEYR,RIDRETH1,DMDBORN2 AS DMDBORN4,DMDEDUC3,DMDEDUC2,DMDMARTL,INDFMPIR,WTMEC2YR,SDMVPSU,SDMVSTRA

FROM DEMO_E

;'

DEMO_E<-sqldf(q)

DEMO_F[]<- lapply(DEMO_F, as.numeric)

q<-'SELECT SEQN,RIAGENDR,RIDAGEYR,RIDRETH1,DMDBORN2 AS DMDBORN4,DMDEDUC3,DMDEDUC2,DMDMARTL,INDFMPIR,WTMEC2YR,SDMVPSU,SDMVSTRA

FROM DEMO_F

;'

DEMO_F<-sqldf(q)

DEMO_G[]<- lapply(DEMO_G, as.numeric)

q<-'SELECT SEQN,RIAGENDR,RIDAGEYR,RIDRETH1,DMDBORN4,DMDEDUC3,DMDEDUC2,DMDMARTL,INDFMPIR,WTMEC2YR,SDMVPSU,SDMVSTRA

FROM DEMO_G

;'

DEMO_G<-sqldf(q)

DEMO_H[]<- lapply(DEMO_H, as.numeric)

q<-'SELECT SEQN,RIAGENDR,RIDAGEYR,RIDRETH1,DMDBORN4,DMDEDUC3,DMDEDUC2,DMDMARTL,INDFMPIR,WTMEC2YR,SDMVPSU,SDMVSTRA

FROM DEMO_H

;'

DEMO_H<-sqldf(q)

DEMO_I[]<- lapply(DEMO_I, as.numeric)

q<-'SELECT SEQN,RIAGENDR,RIDAGEYR,RIDRETH1,DMDBORN4,DMDEDUC3,DMDEDUC2,DMDMARTL,INDFMPIR,WTMEC2YR,SDMVPSU,SDMVSTRA

FROM DEMO_I

;'

DEMO_I<-sqldf(q)

demo<-rbind(DEMO_D,DEMO_E,DEMO_F,DEMO_G,DEMO_H,DEMO_I)

#depression-questionaire

DPQ_D[]<- lapply(DPQ_D, as.numeric)

q<-'SELECT SEQN,DPQ010,DPQ020,DPQ030,DPQ040,DPQ050,DPQ060,DPQ070,DPQ080,DPQ090,DPQ100

FROM DPQ_D

;'

DPQ_D<-sqldf(q)

DPQ_E[]<- lapply(DPQ_E, as.numeric)

q<-'SELECT SEQN,DPQ010,DPQ020,DPQ030,DPQ040,DPQ050,DPQ060,DPQ070,DPQ080,DPQ090,DPQ100

FROM DPQ_E

;'

DPQ_E<-sqldf(q)

DPQ_F[]<- lapply(DPQ_F, as.numeric)

q<-'SELECT SEQN,DPQ010,DPQ020,DPQ030,DPQ040,DPQ050,DPQ060,DPQ070,DPQ080,DPQ090,DPQ100

FROM DPQ_F

;'

DPQ_F<-sqldf(q)

DPQ_G[]<- lapply(DPQ_G, as.numeric)

q<-'SELECT SEQN,DPQ010,DPQ020,DPQ030,DPQ040,DPQ050,DPQ060,DPQ070,DPQ080,DPQ090,DPQ100

FROM DPQ_G

;'

DPQ_G<-sqldf(q)

DPQ_H[]<- lapply(DPQ_H, as.numeric)

q<-'SELECT SEQN,DPQ010,DPQ020,DPQ030,DPQ040,DPQ050,DPQ060,DPQ070,DPQ080,DPQ090,DPQ100

FROM DPQ_H

;'

DPQ_H<-sqldf(q)

DPQ_I[]<- lapply(DPQ_I, as.numeric)

q<-'SELECT SEQN,DPQ010,DPQ020,DPQ030,DPQ040,DPQ050,DPQ060,DPQ070,DPQ080,DPQ090,DPQ100

FROM DPQ_I

;'

DPQ_I<-sqldf(q)

dpq<-rbind(DPQ_D,DPQ_E,DPQ_F,DPQ_G,DPQ_H,DPQ_I)

dpq$dpqflag<-1 #dpq set up new var dpqflag

#questionaire data-reproductive data

RHQ_D[]<- lapply(RHQ_D, as.numeric)

q<-'SELECT SEQN,RHQ010, RHQ031

FROM RHQ_D

;'

RHQ_D<-sqldf(q)

RHQ_E[]<- lapply(RHQ_E, as.numeric)

q<-'SELECT SEQN,RHQ010, RHQ031

FROM RHQ_E

;'

RHQ_E<-sqldf(q)

RHQ_F[]<- lapply(RHQ_F, as.numeric)

q<-'SELECT SEQN,RHQ010, RHQ031

FROM RHQ_F

;'

RHQ_F<-sqldf(q)

RHQ_G[]<- lapply(RHQ_G, as.numeric)

q<-'SELECT SEQN,RHQ010, RHQ031

FROM RHQ_G

;'

RHQ_G<-sqldf(q)

RHQ_H[]<- lapply(RHQ_H, as.numeric)

q<-'SELECT SEQN,RHQ010, RHQ031

FROM RHQ_H

;'

RHQ_H<-sqldf(q)

RHQ_I[]<- lapply(RHQ_I, as.numeric)

q<-'SELECT SEQN,RHQ010, RHQ031

FROM RHQ_I

;'

RHQ_I<-sqldf(q)

rhq<-rbind(RHQ_D,RHQ_E,RHQ_F,RHQ_G,RHQ_H,RHQ_I)

rhq$rhqflag<-1 #rhq set up new var rhqflag

#questionaire data-smoking-cigarette use

SMQ_D[]<- lapply(SMQ_D, as.numeric)

q<-'SELECT SEQN,SMQ020,SMQ040

FROM SMQ_D

;'

SMQ_D<-sqldf(q)

SMQ_E[]<- lapply(SMQ_E, as.numeric)

q<-'SELECT SEQN,SMQ020,SMQ040

FROM SMQ_E

;'

SMQ_E<-sqldf(q)

SMQ_F[]<- lapply(SMQ_F, as.numeric)

q<-'SELECT SEQN,SMQ020,SMQ040

FROM SMQ_F

;'

SMQ_F<-sqldf(q)

SMQ_G[]<- lapply(SMQ_G, as.numeric)

q<-'SELECT SEQN,SMQ020,SMQ040

FROM SMQ_G

;'

SMQ_G<-sqldf(q)

SMQ_H[]<- lapply(SMQ_H, as.numeric)

q<-'SELECT SEQN,SMQ020,SMQ040

FROM SMQ_H

;'

SMQ_H<-sqldf(q)

SMQ_I[]<- lapply(SMQ_I, as.numeric)

q<-'SELECT SEQN,SMQ020,SMQ040

FROM SMQ_I

;'

SMQ_I<-sqldf(q)

smq<-rbind(SMQ_D,SMQ_E,SMQ_F,SMQ_G,SMQ_H,SMQ_I)

#examination data-body measures

BMX_D[]<- lapply(BMX_D, as.numeric)

q<-'SELECT SEQN,BMXBMI

FROM BMX_D

;'

BMX_D<-sqldf(q)

BMX_E[]<- lapply(BMX_E, as.numeric)

q<-'SELECT SEQN,BMXBMI

FROM BMX_E

;'

BMX_E<-sqldf(q)

BMX_F[]<- lapply(BMX_F, as.numeric)

q<-'SELECT SEQN,BMXBMI

FROM BMX_F

;'

BMX_F<-sqldf(q)

BMX_G[]<- lapply(BMX_G, as.numeric)

q<-'SELECT SEQN,BMXBMI

FROM BMX_G

;'

BMX_G<-sqldf(q)

BMX_H[]<- lapply(BMX_H, as.numeric)

q<-'SELECT SEQN,BMXBMI

FROM BMX_H

;'

BMX_H<-sqldf(q)

BMX_I[]<- lapply(BMX_I, as.numeric)

q<-'SELECT SEQN,BMXBMI

FROM BMX_I

;'

BMX_I<-sqldf(q)

bmx<-rbind(BMX_D,BMX_E,BMX_F,BMX_G,BMX_H,BMX_I)

bmx$bmxflag<-1 #bmx set up new var bmxflag

#questionaire data-medical condition

MCQ_D[]<- lapply(MCQ_D, as.numeric)

q<-'SELECT SEQN,MCQ160A,MCQ160B,MCQ160C,MCQ160D,MCQ160E,MCQ160F,MCQ160M,MCQ220,MCQ230A,MCQ230B,MCQ230C,MCQ230D

FROM MCQ_D

;'

MCQ_D<-sqldf(q)

MCQ_E[]<- lapply(MCQ_E, as.numeric)

q<-'SELECT SEQN,MCQ160a,MCQ160b,MCQ160c,MCQ160d,MCQ160e,MCQ160f,MCQ160m,MCQ220,MCQ230a,MCQ230b,MCQ230c,MCQ230d

FROM MCQ_E

;'

MCQ_E<-sqldf(q)

MCQ_F[]<- lapply(MCQ_F, as.numeric)

q<-'SELECT SEQN,MCQ160a,MCQ160b,MCQ160c,MCQ160d,MCQ160e,MCQ160f,MCQ160m,MCQ220,MCQ230a,MCQ230b,MCQ230c,MCQ230d

FROM MCQ_F

;'

MCQ_F<-sqldf(q)

MCQ_G[]<- lapply(MCQ_G, as.numeric)

q<-'SELECT SEQN,MCQ160a,MCQ160b,MCQ160c,MCQ160d,MCQ160e,MCQ160f,MCQ160m,MCQ220,MCQ230a,MCQ230b,MCQ230c,MCQ230d

FROM MCQ_G

;'

MCQ_G<-sqldf(q)

MCQ_H[]<- lapply(MCQ_H, as.numeric)

q<-'SELECT SEQN,MCQ160a,MCQ160b,MCQ160c,MCQ160d,MCQ160e,MCQ160f,MCQ160m,MCQ220,MCQ230a,MCQ230b,MCQ230c,MCQ230d

FROM MCQ_H

;'

MCQ_H<-sqldf(q)

MCQ_I[]<- lapply(MCQ_I, as.numeric)

q<-'SELECT SEQN,MCQ160a,MCQ160b,MCQ160c,MCQ160d,MCQ160e,MCQ160f,MCQ160m,MCQ220,MCQ230a,MCQ230b,MCQ230c,MCQ230d

FROM MCQ_I

;'

MCQ_I<-sqldf(q)

mcq<-rbind(MCQ_D,MCQ_E,MCQ_F,MCQ_G,MCQ_H,MCQ_I)

mcq$mcqflag<-1

#questioniaire-diabete

DIQ_D[]<- lapply(DIQ_D, as.numeric)

q<-'SELECT SEQN, DIQ010

FROM DIQ_D

;'

DIQ_D<-sqldf(q)

DIQ_E[]<- lapply(DIQ_E, as.numeric)

q<-'SELECT SEQN, DIQ010

FROM DIQ_E

;'

DIQ_E<-sqldf(q)

DIQ_F[]<- lapply(DIQ_F, as.numeric)

q<-'SELECT SEQN, DIQ010

FROM DIQ_F

;'

DIQ_F<-sqldf(q)

DIQ_G[]<- lapply(DIQ_G, as.numeric)

q<-'SELECT SEQN, DIQ010

FROM DIQ_G

;'

DIQ_G<-sqldf(q)

DIQ_H[]<- lapply(DIQ_H, as.numeric)

q<-'SELECT SEQN, DIQ010

FROM DIQ_H

;'

DIQ_H<-sqldf(q)

DIQ_I[]<- lapply(DIQ_I, as.numeric)

q<-'SELECT SEQN, DIQ010

FROM DIQ_I

;'

DIQ_I<-sqldf(q)

diq<-rbind(DIQ_D,DIQ_E,DIQ_F,DIQ_G,DIQ_H,DIQ_I)

#Merge together

q<-'SELECT *

FROM demo

LEFT OUTER JOIN dpq ON demo.SEQN=dpq.SEQN

LEFT OUTER JOIN rhq ON demo.SEQN=rhq.SEQN

LEFT OUTER JOIN smq ON demo.SEQN=smq.SEQN

LEFT OUTER JOIN bmx ON demo.SEQN=bmx.SEQN

LEFT OUTER JOIN mcq ON demo.SEQN=mcq.SEQN

LEFT OUTER JOIN diq ON demo.SEQN=diq.SEQN

;'

dat<-sqldf(q)

#x-age of menarche early_menarche=2;normal=1;late_menarche=3

dat$menage[dat$RHQ010>=6 & dat$RHQ010<=11]<-2

dat$menage[dat$RHQ010>=12 & dat$RHQ010<=13]<-1

dat$menage[dat$RHQ010>=14 & dat$RHQ010<=25]<-3

#table(dat$menage[dat$rhqflag %in% c(1)],useNA = "ifany")

#table(dat$menage,useNA = "ifany")

#table(dat$RHQ010,useNA = "ifany")

#y-depression

#table(dat$dpq,useNA = "ifany")

dat$dpq1[dat$DPQ010 %in% c(0)]<-0

dat$dpq1[dat$DPQ010 %in% c(1)]<-1

dat$dpq1[dat$DPQ010 %in% c(2)]<-2

dat$dpq1[dat$DPQ010 %in% c(3)]<-3

dat$dpq1[dat$DPQ010 %in% c(7,9)]<-NA

dat$dpq2[dat$DPQ020 %in% c(0)]<-0

dat$dpq2[dat$DPQ020 %in% c(1)]<-1

dat$dpq2[dat$DPQ020 %in% c(2)]<-2

dat$dpq2[dat$DPQ020 %in% c(3)]<-3

dat$dpq2[dat$DPQ020 %in% c(7,9)]<-NA

dat$dpq3[dat$DPQ030 %in% c(0)]<-0

dat$dpq3[dat$DPQ030 %in% c(1)]<-1

dat$dpq3[dat$DPQ030 %in% c(2)]<-2

dat$dpq3[dat$DPQ030 %in% c(3)]<-3

dat$dpq3[dat$DPQ030 %in% c(7,9)]<-NA

dat$dpq4[dat$DPQ040 %in% c(0)]<-0

dat$dpq4[dat$DPQ040 %in% c(1)]<-1

dat$dpq4[dat$DPQ040 %in% c(2)]<-2

dat$dpq4[dat$DPQ040 %in% c(3)]<-3

dat$dpq4[dat$DPQ040 %in% c(7,9)]<-NA

dat$dpq5[dat$DPQ050 %in% c(0)]<-0

dat$dpq5[dat$DPQ050 %in% c(1)]<-1

dat$dpq5[dat$DPQ050 %in% c(2)]<-2

dat$dpq5[dat$DPQ050 %in% c(3)]<-3

dat$dpq5[dat$DPQ050 %in% c(7,9)]<-NA

dat$dpq6[dat$DPQ060 %in% c(0)]<-0

dat$dpq6[dat$DPQ060 %in% c(1)]<-1

dat$dpq6[dat$DPQ060 %in% c(2)]<-2

dat$dpq6[dat$DPQ060 %in% c(3)]<-3

dat$dpq6[dat$DPQ060 %in% c(7,9)]<-NA

dat$dpq7[dat$DPQ070 %in% c(0)]<-0

dat$dpq7[dat$DPQ070 %in% c(1)]<-1

dat$dpq7[dat$DPQ070 %in% c(2)]<-2

dat$dpq7[dat$DPQ070 %in% c(3)]<-3

dat$dpq7[dat$DPQ070 %in% c(7,9)]<-NA

dat$dpq8[dat$DPQ080 %in% c(0)]<-0

dat$dpq8[dat$DPQ080 %in% c(1)]<-1

dat$dpq8[dat$DPQ080 %in% c(2)]<-2

dat$dpq8[dat$DPQ080 %in% c(3)]<-3

dat$dpq8[dat$DPQ080 %in% c(7,9)]<-NA

dat$dpq9[dat$DPQ090 %in% c(0)]<-0

dat$dpq9[dat$DPQ090 %in% c(1)]<-1

dat$dpq9[dat$DPQ090 %in% c(2)]<-2

dat$dpq9[dat$DPQ090 %in% c(3)]<-3

dat$dpq9[dat$DPQ090 %in% c(7,9)]<-NA

dat$dpqtotal<-dat$dpq1+dat$dpq2+dat$dpq3+dat$dpq4+dat$dpq5+dat$dpq6+dat$dpq7+dat$dpq8+dat$dpq9

dat$dep[dat$dpqtotal<10]<-0

dat$dep[dat$dpqtotal>=10]<-1

dat$miss<-is.na(dat$dpq1)+is.na(dat$dpq2)+is.na(dat$dpq3)+is.na(dat$dpq4)+is.na(dat$dpq5)+is.na(dat$dpq6)+is.na(dat$dpq7)+is.na(dat$dpq8)+is.na(dat$dpq9)

dat$dep[dat$miss %in% c(0) & dat$dpqtotal<=9]<-0

dat$dep[dat$miss %in% c(0) & dat$dpqtotal>=10]<-1

dat$dep[dat$miss %in% c(1) & dat$dpqtotal<=6]<-0

dat$dep[dat$miss %in% c(1) & dat$dpqtotal>=10]<-1

dat$dep[dat$miss %in% c(2) & dat$dpqtotal<=3]<-0

dat$dep[dat$miss %in% c(2) & dat$dpqtotal>=10]<-1

dat$dep[dat$miss %in% c(3) & dat$dpqtotal %in% c(0)]<-0

dat$dep[dat$miss %in% c(3) & dat$dpqtotal>=10]<-1

dat$dep[dat$miss>=4 & dat$dpqtotal>=10]<-1

#table(dat$dep[dat$dpqflag %in% c(1)],useNA = "ifany")

#table(dat$dep,useNA = "ifany")

#AGE

dat$ageg<-7

dat$ageg[dat$RIDAGEYR>=18 & dat$RIDAGEYR<=29]<-1

dat$ageg[dat$RIDAGEYR>=30 & dat$RIDAGEYR<=39]<-2

dat$ageg[dat$RIDAGEYR>=40 & dat$RIDAGEYR<=49]<-3

dat$ageg[dat$RIDAGEYR>=50 & dat$RIDAGEYR<=59]<-4

dat$ageg[dat$RIDAGEYR>=60 & dat$RIDAGEYR<=69]<-5

dat$ageg[dat$RIDAGEYR>=70 & dat$RIDAGEYR<=85]<-6

#table(dat2$ageg,useNA = "ifany")

#RACE, 1=white, 2=black, 3=others

dat$race[dat$RIDRETH1 %in% c(3)]<-1

dat$race[dat$RIDRETH1 %in% c(4)]<-2

dat$race[dat$RIDRETH1 %in% c(1,2,5)]<-3

#table(dat$RIDRETH1,useNA = "ifany")

#EDUCATION, 1=<hs, 2=hs, 3=>hs

dat$edu<-4

dat$edu[dat$DMDEDUC3>=0 & dat$DMDEDUC3<=12]<-1

dat$edu[dat$DMDEDUC3 %in% c(55,66)]<-1

dat$edu[dat$DMDEDUC2 %in% c(1,2)]<-1

dat$edu[dat$DMDEDUC3 %in% c(13,14)]<-2

dat$edu[dat$DMDEDUC2 %in% c(3)]<-2

dat$edu[dat$DMDEDUC3 %in% c(15)]<-3

dat$edu[dat$DMDEDUC2 %in% c(4,5)]<-3

#table(dat$DMDEDUC2,useNA = "ifany")

#table(dat$DMDEDUC3,useNA = "ifany")

#MARITAL, 1=yes, 0=no

dat$marry<-3

dat$marry[dat$DMDMARTL %in% c(1)]<-1

dat$marry[dat$DMDMARTL %in% c(2,3,4,5,6)]<-2

dat$marry2[dat$DMDMARTL %in% c(1)]<-1

dat$marry2[dat$DMDMARTL %in% c(2,3,4,5,6)]<-0

#table(dat$DMDMARTL,useNA = "ifany")

#PIR, 1=<1, 2=1-2, 3=>=2

dat$pir<-4

dat$pir[dat$INDFMPIR>=0 & dat$INDFMPIR<1]<-1

dat$pir[dat$INDFMPIR>=1 & dat$INDFMPIR<2]<-2

dat$pir[dat$INDFMPIR>=2]<-3

#table(dat$INDFMPIR,useNA = "ifany")

#medical condition

#arthritis

dat$arth[dat$MCQ160A %in% c(1)]<-1

dat$arth[dat$MCQ160A %in% c(2)]<-0

#table(dat$arth, useNA = "ifany")

#congestive heart failure

dat$chf[dat$MCQ160B %in% c(1)]<-1

dat$chf[dat$MCQ160B %in% c(2)]<-0

#table(dat$chf, useNA = "ifany")

#coronary heart disease

dat$chd[dat$MCQ160C %in% c(1)]<-1

dat$chd[dat$MCQ160C %in% c(2)]<-0

#table(dat$chd, useNA = "ifany")

#angina

dat$angi[dat$MCQ160D %in% c(1)]<-1

dat$angi[dat$MCQ160D %in% c(2)]<-0

#table(dat$angi, useNA = "ifany")

#heart attack

dat$hat[dat$MCQ160E %in% c(1)]<-1

dat$hat[dat$MCQ160E %in% c(2)]<-0

#table(dat$hat, useNA = "ifany")

#stroke

dat$strok[dat$MCQ160F %in% c(1)]<-1

dat$strok[dat$MCQ160F %in% c(2)]<-0

#table(dat$strok, useNA = "ifany")

#cvd-all

dat$cvd[dat$MCQ160B %in% c(1) | dat$MCQ160C %in% c(1) | dat$MCQ160D %in% c(1) | dat$MCQ160E %in% c(1) | dat$MCQ160F %in% c(1)]<-1

dat$cvd[dat$MCQ160B %in% c(2) & dat$MCQ160C %in% c(2) & dat$MCQ160D %in% c(2) & dat$MCQ160E %in% c(2) & dat$MCQ160F %in% c(2)]<-0

#table(dat$cvd, useNA ="ifany")

#thyroid

dat$thyr[dat$MCQ160M %in% c(1)]<-1

dat$thyr[dat$MCQ160M %in% c(2)]<-0

#table(dat$thyr, useNA = "ifany")

#cancer

dat$cance[dat$MCQ220 %in% c(1)]<-1

dat$cance[dat$MCQ220 %in% c(2)]<-0

#table(dat$cance, useNA = "ifany")

#diabetes

dat$diabe[dat$DIQ010 %in% c(1,3)]<-1

dat$diabe[dat$DIQ010 %in% c(2)]<-0

#table(dat$diabe, useNA = "ifany")

#breast cancer

dat$brtcan[dat$MCQ220 %in% c(1,2)]<-0

dat$brtcan[(dat$MCQ220 %in% c(1)) & ((dat$MCQ230A %in% c(14))|(dat$MCQ230B %in% c(14))|(dat$MCQ230C %in% c(14))|(dat$MCQ230D %in% c(14)))]<-1

#table(dat$brtcan, useNA = "ifany")

#smoking-1=CURRENT SMOKER, 2=FORMER SMOKER, 3=NEVER SMOKER

dat$smoke<-4

dat$smoke[dat$SMQ020 %in% c(2)]<-3

dat$smoke[dat$SMQ020 %in% c(1) & dat$SMQ040 %in% c(3) ]<-2

dat$smoke[dat$SMQ020 %in% c(1) & dat$SMQ040 %in% c(1,2) ]<-1

#regular periods in the past 12 months

dat$rp<-2

dat$rp[dat$RHQ031 %in% c(1)]<-1

dat$rp[dat$RHQ031 %in% c(2)]<-0

#table(dat$SMQ040, useNA = "ifany")

#bmi(current) 1: <18.5 2: 18.5-25.0 3: 25-30 4: 30-35 5: 35+ 6: MISSING;

dat$bmig[dat$BMXBMI<18.5 & dat$BMXBMI>0]<-1

dat$bmig[dat$BMXBMI>=18.5 & dat$BMXBMI<25]<-2

dat$bmig[dat$BMXBMI>=25 & dat$BMXBMI<30]<-3

dat$bmig[dat$BMXBMI>=30 & dat$BMXBMI<35]<-4

dat$bmig[dat$BMXBMI>=35]<-5

dat$bmig[dat$BMXBMI %in% c(NA)]<-6

#table(dat$bmig,useNA = "ifany")

#table(dat$bmig[dat$bmxflag %in% c(1)], useNA = "ifany")

#Weight

dat$weight<-dat$WTMEC2YR/6

#Exclusion

dat2e<-dat[dat$rhqflag %in% c(1) & (is.na(dat$dep)|is.na(dat$menage)),]

dat2e$ageg[dat2e$ageg %in% c(7)]<-1

dat2ea<-dat[dat$rhqflag %in% c(1) & (is.na(dat$dep)|is.na(dat$menage)) & (dat$RIDAGEYR>=18),]

temp<-dat[(!is.na(dat$rhqflag))& (dat$RIAGENDR %in% c(2)) & (dat$RIDAGEYR>=18) ,]

temp<-dat[(!is.na(dat$rhqflag))& (dat$RIAGENDR %in% c(2)) & (dat$RIDAGEYR>=18) &(!is.na(dat$menage))&(!is.na(dat$dep)),]

dat2<-dat[(!is.na(dat$dep))&(!is.na(dat$menage)),]

#table1

CreateCatTable(vars = c("menage","ageg","race","edu","pir","marry","bmig","smoke","rp"),data = dat2,includeNA = T)

CreateCatTable(vars = c("menage","ageg","race","edu","pir","marry","bmig","smoke","rp"),strata = "dep",data = dat2,includeNA = T)

design <- svydesign(id = ~ SDMVPSU, strata = ~ SDMVSTRA, nest = TRUE, weight = ~ weight, data = dat2)

svyCreateCatTable(vars = c("menage","ageg","race","edu","pir","marry","bmig","smoke","rp"),data = design,includeNA = T)

svyCreateCatTable(vars = c("menage","ageg","race","edu","pir","marry","bmig","smoke","rp"),strata = "dep",data = design,includeNA = T)

#weighted CI

confint(svyciprop(~I(rp %in% c(0)), design, method="logit"))

#survey design for svyproportion-table1-weighted CI

#dep=1, calcualte covariates

dat3<-dat2[dat2$dep %in% c(1),]

design <- svydesign(id = ~ SDMVPSU, strata = ~ SDMVSTRA, nest = TRUE, weight = ~ weight, data = dat3)

(a<-svyciprop(~I(rp %in% c(NA)), design, method="logit"))

confint(a)

#dep=0, calculate covariates

dat3<-dat2[dat2$dep %in% c(0),]

design <- svydesign(id = ~ SDMVPSU, strata = ~ SDMVSTRA, nest = TRUE, weight = ~ weight, data = dat3)

(a<-svyciprop(~I(rp %in% c(NA)), design, method="logit"))

confint(a)

#logistic regression models

#survey design for logistic regression

#survey design

design <- svydesign(id = ~ SDMVPSU, strata = ~ SDMVSTRA, nest = TRUE, weight = ~ weight, data = dat2)

#original models

model1<-svyglm(dep ~ factor(menage), design = design, family=quasibinomial())

cbind(exp(coef(model1)),exp(confint(model1)))

model2<-svyglm(dep ~ factor(menage)+factor(ageg)+factor(race)+factor(edu)+factor(pir)+factor(marry), design = design, family=quasibinomial())

cbind(exp(coef(model2)),exp(confint(model2)))

model3<-svyglm(dep ~ factor(menage)+factor(ageg)+factor(race)+factor(edu)+factor(pir)+factor(marry)+factor(bmig)+factor(smoke)+factor(rp), design = design, family=quasibinomial())

round(cbind(exp(coef(model3)),exp(confint(model3))),2)

#continuous age at menarche

model1<-svyglm(dep ~ RHQ010, design = design, family=quasibinomial())

1/cbind(exp(coef(model1)),exp(confint(model1)))

model2<-svyglm(dep ~ RHQ010+factor(ageg)+factor(race)+factor(edu)+factor(pir)+factor(marry), design = design, family=quasibinomial())

1/cbind(exp(coef(model2)),exp(confint(model2)))

cbind(exp(coef(model2)),exp(confint(model2)))

model3<-svyglm(dep ~ RHQ010+factor(ageg)+factor(race)+factor(edu)+factor(pir)+factor(marry)+factor(bmig)+factor(smoke)+factor(rp), design = design, family=quasibinomial())

1/cbind(exp(coef(model3)),exp(confint(model3)))

round(cbind(exp(coef(model3)),exp(confint(model3))),2)

#interaction between age

model1<-svyglm(dep ~ factor(menage)*factor(ageg), design = design, family=quasibinomial())

cbind(exp(coef(model1)),exp(confint(model1)))

model2<-svyglm(dep ~ factor(menage)*factor(ageg)+factor(race)+factor(edu)+factor(pir)+factor(marry), design = design, family=quasibinomial())

summary(model2)

cbind(exp(coef(model2)),exp(confint(model2)))[2:3,]

model3<-svyglm(dep ~ factor(menage)*factor(ageg)+factor(race)+factor(edu)+factor(pir)+factor(marry)+factor(bmig)+factor(smoke)+factor(rp), design = design, family=quasibinomial())

summary(model3)

cbind(exp(coef(model3)),exp(confint(model3)))[2:3,]

#multiple imputation

mdat<-dat[(!is.na(dat$rhqflag))&(!is.na(dat$dpqflag))&(dat$RIDAGEYR>=18)&(dat$RIAGENDR %in% c(2)),]

mdat$dep<-as.factor(mdat$dep)

mdat$menage<-as.factor(mdat$menage)

mdat$ageg<-as.factor(mdat$ageg)

mdat$race<-as.factor(mdat$race)

mdat$edu<-as.factor(mdat$edu)

mdat$pir<-as.factor(mdat$pir)

mdat$marry<-as.factor(mdat$marry)

mdat$bmig<-as.factor(mdat$bmig)

mdat$smoke<-as.factor(mdat$smoke)

mdat$rp<-as.factor(mdat$rp)

#categorical

mdat2<-mdat[,c("dep","menage","ageg","race","edu","pir","marry","bmig","smoke","rp")]

imp<-mice(data = mdat2,m = 50)

resm3<-data.frame(est1=numeric(),se1=numeric(),est2=numeric(),se2=numeric())

for (i in 1:50){

impdata<-complete(imp,i)

impdata<-cbind(impdata,mdat[,c("SDMVPSU","SDMVSTRA","weight")])

design <- svydesign(id = ~ SDMVPSU, strata = ~ SDMVSTRA, nest = TRUE, weight = ~ weight, data = impdata)

model3<-svyglm(dep ~ factor(menage)+factor(ageg)+factor(race)+factor(edu)+factor(pir)+factor(marry)+factor(bmig)+factor(smoke)+factor(rp), design = design, family=quasibinomial())

resm3[i,1]<-model3$coefficients[2]

resm3[i,2]<-SE(model3)[2]

resm3[i,3]<-model3$coefficients[3]

resm3[i,4]<-SE(model3)[3]

print(i)

}

c(exp(mean(resm3$est1)),exp(mean(resm3$est1)-1.96*mean(resm3$se1)),exp(mean(resm3$est1)+1.96*mean(resm3$se1)))

c(exp(mean(resm3$est2)),exp(mean(resm3$est2)-1.96*mean(resm3$se2)),exp(mean(resm3$est2)+1.96*mean(resm3$se2)))

#continuous

mdat2<-mdat[,c("dep","RHQ010","ageg","race","edu","pir","marry","bmig","smoke","rp")]

imp<-mice(data = mdat2,m = 50)

resm3<-data.frame(est1=numeric(),se1=numeric())

for (i in 1:50){

impdata<-complete(imp,i)

impdata<-cbind(impdata,mdat[,c("SDMVPSU","SDMVSTRA","weight")])

design <- svydesign(id = ~ SDMVPSU, strata = ~ SDMVSTRA, nest = TRUE, weight = ~ weight, data = impdata)

model3<-svyglm(dep ~ RHQ010+factor(ageg)+factor(race)+factor(edu)+factor(pir)+factor(marry)+factor(bmig)+factor(smoke)+factor(rp), design = design, family=quasibinomial())

resm3[i,1]<-model3$coefficients[2]

resm3[i,2]<-SE(model3)[2]

print(i)

}

c(1/exp(mean(resm3$est1)),1/exp(mean(resm3$est1)-1.96*mean(resm3$se1)),1/exp(mean(resm3$est1)+1.96*mean(resm3$se1)))

#alternatively

for (i in 1:50){

assign(paste0("imp",i),cbind(complete(imp,i),mdat[,c("SDMVPSU","SDMVSTRA","weight")]))

print(i)

}

il<-imputationList(datasets = list(imp1,imp2,imp3,imp4,imp5,imp6,imp7,imp8,imp9,imp10,imp11,imp12,imp13,imp14,imp15,imp16,imp17,imp18,imp19,imp20,imp21,imp22,imp23,imp24,imp25,imp26,imp27,imp28,imp29,imp30,imp31,imp32,imp33,imp34,imp35,imp36,imp37,imp38,imp39,imp40,imp41,imp42,imp43,imp44,imp45,imp46,imp47,imp48,imp49,imp50))

designs <- svydesign(id = ~ SDMVPSU, strata = ~ SDMVSTRA, nest = TRUE, weight = ~ weight, data = il)

results<-with(designs, svyglm(dep ~ RHQ010+factor(ageg)+factor(race)+factor(edu)+factor(pir)+factor(marry)+factor(bmig)+factor(smoke)+factor(rp), family=quasibinomial()))

1/exp(MIcombine(results)$coefficients[2])

1/exp(MIcombine(results)$coefficients[2]-1.96*SE(MIcombine(results))[2])

1/exp(MIcombine(results)$coefficients[2]+1.96*SE(MIcombine(results))[2])
